# Hybrid deep learning and optimized variational mode decomposition for point-interval runoff prediction

**DOI:** 10.1371/journal.pone.0343063

**Published:** 2026-03-17

**Authors:** Hong Ma, Muhammad Fadhil Marsani, Mohd. Asyraf Mansor, Mohd Shareduwan Mohd Kasihmuddin

**Affiliations:** 1 School of Financial Mathematics and Statistics, Guangdong University of Finance, Guangzhou, China; 2 School of Mathematical Sciences, Universiti Sains Malaysia, Penang, Malaysia; 3 School of Distance Education, Universiti Sains Malaysia, Penang, Malaysia; Swedish Meteorological and Hydrological Institute, SWEDEN

## Abstract

Runoff prediction is crucial for water resource allocation and hydropower planning. To address low accuracy and uncertainty in runoff forecasting, this study proposes a framework integrating the Information Acquisition Optimizer (IAO), Variational Mode Decomposition (VMD), Convolutional Neural Network-Support Vector Machine (CNN-SVM), and Kernel Density Estimation (KDE) for interval prediction. An IAO-based optimized VMD (IVMD) is employed to decompose non-stationary runoff series and enhance feature extraction, with the resulting components used as inputs to the CNN-SVM model for point prediction. To quantify predictive uncertainty, KDE is applied to model the prediction error distribution, where a B-spline-based least squares cross-validation bandwidth selection method (LSCV-B) is adopted. By combining B-spline basis functions with data-driven cross-validation, LSCV-B overcomes the limited local adaptability of conventional AMISE-based bandwidth selection, enabling more accurate error density estimation and narrower prediction intervals with reliable coverage. Experiments in the Yangtze River Basin show that the IVMD-CNN-SVM framework reduces RMSE and MAPE by **approximately** 40–50% on the testing dataset compared with VMD-based counterparts, while producing highly reliable and compact 90% interval predictions.

## 1. Introduction

Water resources are fundamental to agricultural production and socioeconomic development, with runoff serving as a critical component of regional hydrological processes. In recent years, global climate change, intensified extreme weather events, and human activities have substantially increased the complexity and variability of runoff dynamics, posing significant challenges to effective water resource management [1,2]. Consequently, accurate runoff prediction and uncertainty quantification have become pressing issues in hydrology and environmental science [3].

Traditional time series forecasting methods mainly include statistical techniques such as time series analysis, autoregressive moving average models (ARIMA), and multiple linear regression [4,5]. Although these methods perform well under certain conditions, they often produce large errors when dealing with nonlinear and non-stationary data, especially during extreme weather and sudden events [6]. With the rapid development of artificial intelligence, machine learning algorithms such as Support Vector Machines (SVM), Random Forests (RF), and Relevance Vector Machines (RVM) have been increasingly applied to runoff forecasting [7]. These algorithms exhibit significant advantages in modeling complex nonlinear relationships and effectively capturing hidden patterns in the data, thereby greatly improving prediction accuracy. Liu et al. [8] proposed a runoff prediction error correction method that combines LSSVM and a four-dimensional copula joint distribution, significantly improving accuracy and reducing flood overestimation. Samantaray and Ghose [9] developed a PSR-SVM-FFA hybrid model for predicting runoff from rainfall simulator experimental datasets, achieving error control within 2% to 3%. Some researchers constructed models using multiple machine learning algorithms and found that SVM generally exhibited higher predictive accuracy than other tested models [10]. However, the spatiotemporal complexity of runoff sequences still poses significant challenges to accurate runoff prediction [11].

Convolutional neural networks have demonstrated strong capability in learning complex spatial and spatiotemporal patterns and have been successfully applied to various prediction tasks involving structured data [12–15]. Considering CNN's ability to extract local and global features from complex spatiotemporal data and SVM’s excellent regression performance, An et al. [16] constructed an SSA-CNN-SVM model for predicting hourly heating loads in residential buildings, achieving a MAPE ranging from 2.28% to 2.4%; after introducing indoor temperature data, MAPE decreased by 0.35%. Bhattacharyya et al. [17] developed a hybrid model integrating GPM, CNN, and SVM for sugarcane yield prediction, achieving an accuracy improvement of 89.53% over traditional methods and significantly enhancing predictions of soil moisture and crop yield. However, a major challenge in runoff prediction lies in the non-stationarity of runoff data, manifested in changing amplitudes, frequencies, and trends [18]. Although machine learning algorithms offer strong adaptability, their direct application to runoff prediction is often affected by residual noise in the data, limiting prediction accuracy.

Preprocessing runoff data can mitigate the impact of noise. As such, researchers have developed stable decomposition-based hybrid forecasting models that integrate signal decomposition with forecasting models, significantly improving runoff prediction accuracy [19–22]. In hydrological forecasting, Variational Mode Decomposition (VMD), as a time-frequency analysis method, effectively decomposes nonlinear components in signals, providing more accurate features for subsequent modeling [23]. However, the performance of VMD strongly depends on the choice of the number of modes and the penalty factor, which in many studies are specified empirically or tuned using conventional optimization strategies, potentially limiting robustness and efficiency [24–26]. To address this issue, a variety of optimization-based strategies have been introduced to improve VMD parameter selection. However, many conventional metaheuristic algorithms still suffer from high computational complexity or slow convergence. Recently, the Information Acquisition Optimizer (IAO), a novel metaheuristic algorithm inspired by the human process of information collection, filtering, and evaluation, has been proposed and shown to improve both the accuracy and efficiency of VMD parameter optimization [27].

However, point forecasts provide deterministic outputs and thus fail to capture runoff uncertainty, and most existing decomposition-based hybrid models pay limited attention to systematic uncertainty quantification [28,29]. Probabilistic interval forecasting, on the other hand, offers not only point predictions but also prediction intervals at a given confidence level, which is more practical for water resource allocation and hydropower planning [30]. Non-parametric Kernel Density Estimation (KDE) estimates the probability density function (PDF) by weighting data points using kernel functions, producing predictive intervals that help decision-makers intuitively understand forecast uncertainty [31–34]. Least Squares Cross-Validation combined with B-spline basis functions (LSCV-B) further improves the accuracy and stability of bandwidth selection in KDE.

Although recent decomposition-ensemble or physics-informed hybrid models have advanced hydrological forecasting, they often use empirical VMD parameters and treat point prediction and uncertainty quantification separately. In contrast, the proposed IVMD-CNN-SVM-LSCV-B framework jointly optimizes decomposition (via IAO), enhances feature-regression synergy (CNN-SVM), and generates coherent prediction intervals (LSCV-B), offering a more integrated and adaptive solution.

The main innovations of this study include:

i By adaptively optimizing the number of modes and the penalty factor, the IAO algorithm substantially enhances VMD decomposition quality, thereby providing more accurate and informative inputs for subsequent prediction tasks.
ii The integration of CNN-based feature learning with SVM regression enables effective representation extraction and stable prediction, thereby enhancing overall model robustness.
iii LSCV-B spline method: The incorporation of LSCV-B for bandwidth selection in KDE improves the precision and reliability of interval forecasting.

Case studies at the Hankou and Luoshan stations in the Yangtze River Basin confirm the effectiveness of the IVMD-CNN-SVM-LSCV-B model in improving runoff prediction accuracy for complex datasets.

## 2. Runoff point prediction techniques

### 2.1. Optimized variational mode decomposition

#### 2.1.1. Information Acquisition Optimizer (IAO).

The Information Acquisition Optimizer (IAO) is a novel metaheuristic algorithm inspired by human cognitive processes of acquiring, filtering, and organizing information to solve complex optimization problems. It operates through an iterative cycle that begins with diverse solution initialization, followed by stochastic perturbation and greedy selection to refine candidates, and finally employs a best-guided adaptive mechanism to accelerate convergence toward global optima. This staged strategy enables IAO to balance exploration and exploitation effectively, while maintaining population diversity and guiding the search toward promising regions of the solution space.

**Fig 1:**
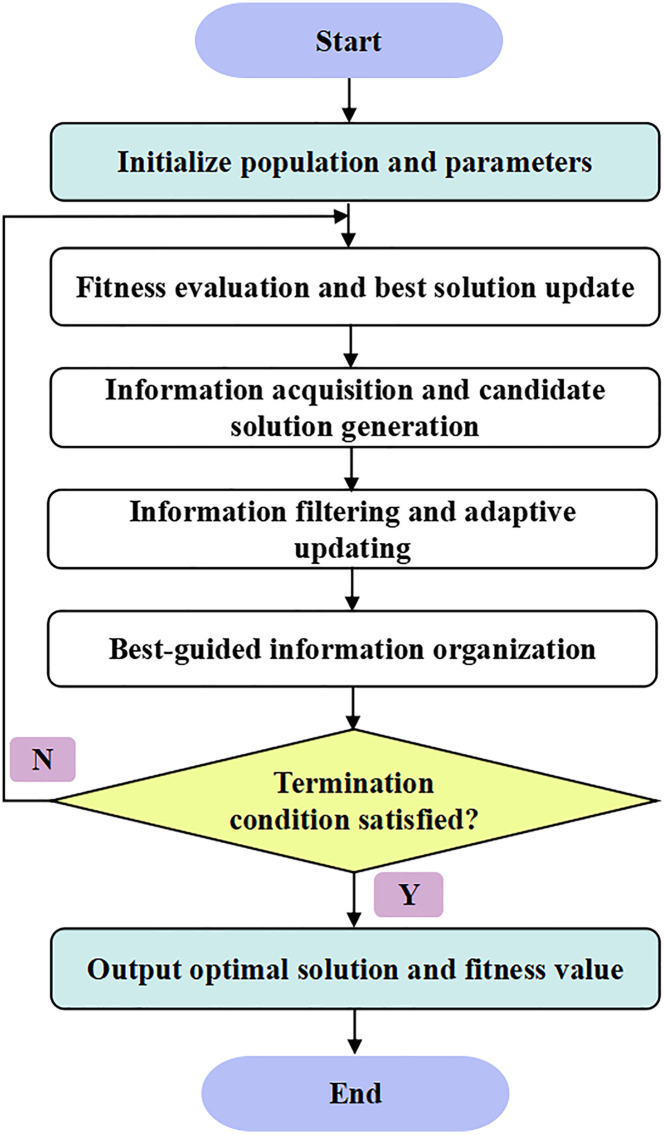
Workflow of the IAO algorithm, including population initialization, information acquisition, fitness evaluation, and best-guided updating.

The complete algorithmic workflow of the proposed method is illustrated in Fig 1, and its core computational components are summarized in Table 1.

**Table 1:** Core components and operational phases of the IAO.

Component	Description	Mathematical/ Algorithmic Form
Initialization	Random population generation within bounds	$X_{i}^{\left(\text{0}\right)}=LB+rand\cdot (UB-LB)$
Candidate Generation	Stochastic differential information acquisition	$X_{i}^{new}=X_{i}+\left(X_{r\text{1}}-X_{r\text{2}}\right)\cdot \theta ,\;\theta \sim u(-\text{1},\text{1})$
Adaptive Filtering	Dynamic coefficient driven neighborhood updating	$\begin{array}{c} \begin{array}{c} \phi =\left(\text{1}+cos\left(\text{2}r_{\text{1}}\right)\right)\left(\text{1}-\frac{t}{T}\right),\mathrm{\backslash vspace}2mm\\ \gamma =sin\left({\left(\frac{\pi }{\text{4}}\right)}^{\frac{t}{T}}\right)+\phi +\frac{ln\left(\frac{t}{T}\right)}{\text{8}},\mathrm{\backslash vspace}2mm\\ \xi =\text{2}\cdot mod\left(\text{3}.\text{4}\text{6}\text{8}\cdot r_{\text{2}}\cdot \left(\text{1}-r_{\text{3}}\cdot cos\left(arccos\left(r_{\text{4}}\cdot {\text{1}\text{0}}^{\text{4}}\right)\right),\text{1}\right)\right),\mathrm{\backslash vspace}2mm\\ \delta =\frac{cos\left(\frac{\pi }{\text{2}}\sqrt{\left|\gamma \right|}\right)}{\xi },\mathrm{\backslash vspace}2mm\\ \lambda ={\text{2}}^{\sqrt{\left|\gamma \right|}-\text{2}} \end{array} \end{array}$
Best-Guided Refinement	Conditional best-guided cosine-modulated updating	$\begin{array}{c} \begin{array}{c} X_{i}^{new}=X_{best}\cdot cos\left(\frac{\pi }{\text{2}}\sqrt[{\text{3}}]{\left|\gamma \right|}\right)-△,\\ where\\ △=\left\{ \begin{array}{c} r_{\text{9}}\left({\overset{―}{X}}_{best}-X_{i}\right),\;if\;\;\phi \geq \text{0}.\text{5}\\ \text{0}.\text{8}\left(r_{\text{1}\text{0}}r_{\text{1}\text{1}}X_{best}-\left(\text{2}r_{\text{1}\text{2}}-\text{1}\right)X_{i}\right),\;otherwise \end{array}\right.\;\\ with\;r_{\text{9}}{,r}_{\text{1}\text{0}},r_{\text{1}\text{1}}{,r}_{\text{1}\text{2}}\sim u(\text{0},\text{1}),\;and\;{\overset{―}{X}}_{best}=mean\left(X_{best}\right). \end{array} \end{array}$
Boundary Handling	Feasible range clamping	$x_{j}=min\left(max\left(x_{j},LB_{j}\right),UB_{j}\right)$

Note: $X_{i}$: position (solution vector) of the i-th agent; $X_{best}$: current best solution; $r_{\text{1}},r_{\text{2}}$: distinct random indices in {1,2,⋯,N}; *t*: current iteration; *T*: maximum number of iterations; All random numbers $r_{k}\sim u(\text{0},\text{1})$ are independently sampled.

#### 2.1.2. Variational mode decomposition (VMD).

Variational Mode Decomposition (VMD), proposed by Dragomiretskiy and Zosso, is a non-recursive signal decomposition method that formulates the decomposition process as a constrained variational optimization problem. The objective of VMD is to decompose a given signal into a predefined number of band-limited intrinsic mode functions (IMFs), each associated with a specific center frequency.

For a real-valued signal f(t), VMD seeks a set of modes ${\left\{u_{k}(t)\right\}}_{k=\text{1}}^{K}$ and their corresponding center frequencies ${\left\{\omega_{k}\right\}}_{k=\text{1}}^{K}$ by minimizing the sum of the bandwidths of all modes. The bandwidth of each mode is measured as the squared $L_{\text{2}}$-norm of the gradient of its analytic signal after frequency shifting. The optimization problem is formulated as


$$\underset{\left\{u_{k}\right\},\left\{\omega_{k}\right\}}{min}\sum_{k=\text{1}}^{K}{\left\|\partial_{t}\left[\left(\delta (t)+\frac{j}{\pi t}\right)*u_{k}(t)\right]e^{-j\omega_{k}t}\right\|}_{\text{2}}^{\text{2}}$$ (1)

subject to the reconstruction constraint


$$\sum_{k=\text{1}}^{K}u_{k}(t)=f(t)$$ (2)

where δ(t) denotes the Dirac delta function, * represents convolution, and *j* is the imaginary unit.

To solve the constrained problem, an augmented Lagrangian is constructed by introducing a quadratic penalty factor α and a Lagrange multiplier λ(t), expressed as


$$L\left(\left\{u_{k}\right\}\;,\left\{\omega_{k}\right\}\;,\lambda \right)=\alpha \sum_{k=\text{1}}^{K}{\left\|\partial_{t}\left[\left(\delta (t)+\frac{j}{\pi t}\right)*u_{k}(t)\right]e^{-j\omega_{k}t}\right\|}_{\text{2}}^{\text{2}}+{\left\|f(t)-\sum_{k=\text{1}}^{K}u_{k}(t)\right\|}_{\text{2}}^{\text{2}}++\left\langle \lambda (t),f(t)-\sum_{k=\text{1}}^{K}u_{k}(t)\right\rangle$$ (3)

The resulting optimization problem is solved iteratively in the frequency domain using the alternating direction method of multipliers (ADMM), where the mode spectra and center frequencies are updated alternately until convergence. After convergence, the decomposed modes are reconstructed in the time domain via inverse Fourier transform.

#### 2.1.3. IAO-VMD (IVMD).

Building upon the basic VMD framework introduced in the previous subsection, this subsection proposes an entropy-guided decomposition framework, termed IAO optimized VMD (IVMD), for complex runoff series. By embedding the IAO into the VMD parameter selection process, IVMD enables automatic and data-driven determination of key decomposition parameters, thereby improving decomposition robustness and reliability when handling non-stationary and highly nonlinear hydrological signals.

The decomposition performance of conventional VMD is highly sensitive to the selection of two key parameters, namely the number of modes *K* and the penalty factor α. In most existing studies, these parameters are determined empirically or through trial-and-error, which often leads to sub-optimal decomposition results and limits the adaptability of VMD to complex hydrological time series characterized by strong non-linearity and non-stationarity. Inappropriate parameter settings may further propagate errors to subsequent prediction models.

To overcome this limitation, this study employs the IAO to automatically optimize *K* and α within a unified optimization framework. The optimization process is guided by Sample Entropy (SE), which serves as a data-driven metric to evaluate decomposition quality without requiring any prior prediction. SE is a widely used complexity measure for time series analysis, quantifying the degree of irregularity and unpredictability in a signal. Lower SE values indicate higher regularity, stronger temporal correlation, and reduced randomness, implying that the underlying dynamics of the signal are more structured and predictable. By minimizing SE, the optimization process encourages the extraction of IMFs with clearer structure and reduced complexity, which is particularly desirable for subsequent modeling and forecasting tasks.

Through entropy-guided optimization of VMD parameters, the proposed IVMD achieves more stable and reliable signal decomposition. The resulting IMFs exhibit enhanced temporal regularity and reduced complexity, facilitating clearer representation of the underlying runoff dynamics. Consequently, IVMD provides higher quality and more informative input features for subsequent deep learning-based runoff forecasting models.

### 2.2. Convolutional Neural Network (CNN)

Convolutional Neural Networks (CNNs) are employed in this study to automatically extract representative features from decomposed runoff components. A one-dimensional CNN architecture is constructed, where the normalized input sequences are arranged as two-dimensional inputs of size N × 1. The CNN consists of two convolutional layers. The first layer uses 16 convolutional kernels with a kernel size of 3 × 1, followed by batch normalization, a ReLU activation function, and a max-pooling layer with a pooling size of 2 × 1 and stride 1. The second convolutional layer employs 32 kernels with a kernel size of 2 × 1, followed by batch normalization, ReLU activation, and an identical max-pooling operation. After feature extraction, two fully connected layers with 25 and 1 neurons, respectively, are used for regression.

The network is trained using the Adam optimizer with a maximum of 150 epochs, an initial learning rate of 0.01, and an L2 regularization coefficient of 0.001. Features extracted from the second pooling layer are subsequently used as inputs to the SVM model for regression.

### 2.3. Support vector machine (SVM)

The Support Vector Machine (SVM) algorithm is a supervised machine learning method widely used for solving complex classification and regression problems. It is particularly effective for handling nonlinear, high-dimensional, and small-sample datasets, offering strong local and global search capabilities.

Rooted in statistical learning theory, SVM was initially developed for classification tasks but can be extended to regression problems known as Support Vector Regression (SVR) through the use of kernel functions. The core idea of SVM is to construct an optimal separating hyperplane that maximally distinguishes between data points of different classes, thereby enabling efficient classification or regression.

In regression tasks, the objective of SVM is to find a function f(x) that approximates the target *y* values as closely as possible while maintaining low model complexity. SVM regression achieves this by introducing an ε-insensitive loss function. Specifically, the goal of SVM regression is to minimize the following objective function:


$$\begin{array}{c} {min}_{w,b,\xi ,\xi^{*}}(\frac{\text{1}}{\text{2}}{\left\|w\right\|}^{\text{2}}+C\sum_{i=\text{1}}^{n}\left(\xi_{i}+{\xi_{i}}^{*}\right)) \end{array}$$ (4)

The corresponding constraints are defined as follows:


$$\left\{ \begin{array}{c} y_{i}-\left\langle \omega ,\phi \left(x_{i}\right)\right\rangle -b\leq \varepsilon +\xi_{i}\\ \left\langle \omega ,\phi \left(x_{i}\right)\right\rangle +b-y_{i}\leq \varepsilon +{\xi_{i}}^{*}\\ \xi_{i}{{,\xi }_{i}}^{*}\geq \text{0},\;i=\text{1},\text{2},\cdots ,n \end{array}\right.\;$$ (5)

where *w* is the normal vector of the hyperplane; *b* is the bias term; $\xi_{i}$ and ${\xi_{i}}^{*}$ are slack variables representing the deviations of the predicted values beyond the ε margin; *C* is the penalty coefficient that controls the trade-off between model complexity and fitting error.

In this study, SVM is employed in the form of SVR to model the nonlinear relationship between CNN-extracted features and runoff values. The feature representations obtained from the second pooling layer of the CNN are used as inputs to the SVR model. A linear kernel function is adopted to reduce model complexity and enhance generalization when handling high-dimensional deep features. The penalty coefficient *C* is set to 0.01, which is determined empirically under normalized feature space to balance regression accuracy and model stability, thereby mitigating the risk of overfitting.

#### 2.3.1. Kernel function of SVM.

By introducing kernel functions, SVM is capable of mapping data into a high-dimensional feature space, thereby effectively handling nonlinear relationships. Commonly used kernel functions include the linear kernel, polynomial kernel, radial basis function (RBF) kernel, and sigmoid kernel, which correspond to Equations (6) through (9), respectively. In this study, the linear kernel was selected for prediction.


$$K(x,x^{\prime })=x^{T}x^{\prime }$$ (6)

$$K(x,x^{\prime })={\left(x^{T}x^{\prime }+\text{1}\right)}^{d}$$ (7)

$$K(x,x^{\prime })=e^{-\gamma {\left\|x-x^{\prime }\right\|}^{\text{2}}}$$ (8)

$$K(x,x^{\prime })=tanh(\alpha x^{T}x^{\prime }+\beta )$$ (9)

## 3. Runoff interval forecasting based on Kernel Density Estimation (KDE)

In runoff forecasting, beyond achieving accurate point predictions, it is equally important to quantify the uncertainty associated with the predicted results. Interval prediction provides upper and lower bounds at a specified confidence level, thereby offering a more comprehensive assessment of prediction reliability and robustness under uncertain hydrological conditions.

Kernel Density Estimation (KDE) is a widely used non-parametric approach for probabilistic modeling and uncertainty quantification. By superimposing kernel functions centered at each observation and averaging their contributions, KDE enables the estimation of the underlying probability density of forecast residuals. However, the effectiveness of KDE is highly dependent on the choice of bandwidth. An excessively small bandwidth may lead to over-fitting and spurious fluctuations, whereas an overly large bandwidth may result in over-smoothing and loss of important distributional features, both of which can adversely affect the accuracy of interval estimates.

To facilitate subsequent interval construction based on KDE, the fundamental principle of kernel density estimation is first introduced in the following subsection.

### 3.1. The fundamental principle of KDE

For a given data sequence ${\left\{x_{i}\right\}}_{i=\text{1}}^{n}$, the KDE is defined as


$$\begin{array}{c} \hat{f}(x)=\frac{\text{1}}{nh}\sum_{i=\text{1}}^{n}K\left(\frac{x-x_{i}}{h}\right) \end{array}$$ (10)

where *n* denotes the number of data points, $x_{i}$ is the i-th observation, *h* represents the bandwidth, and K(·) is the kernel function.

The bandwidth *h* is a critical parameter in KDE, as it directly controls the degree of smoothing of the estimated density function. An excessively small bandwidth may lead to over-fitting, resulting in pronounced local fluctuations and noisy density estimates, whereas an overly large bandwidth may cause over-smoothing, obscuring important structural features of the underlying distribution and reducing estimation accuracy.

In comparison, the choice of kernel function generally has a relatively smaller impact on the estimation performance than the bandwidth. Consequently, bandwidth selection plays a dominant role in density estimation accuracy.

Among commonly used kernel functions, the Gaussian kernel is widely adopted due to its smoothness and numerical stability. Therefore, the Gaussian kernel is employed in this study, and its expression is given by


$$K(u)=\frac{\text{1}}{\sqrt{\text{2}\pi }}e^{-\frac{u^{\text{2}}}{\text{2}}}$$ (11)

### 3.2. LSCV method

Least-squares cross-validation (LSCV) is a widely used bandwidth selection method that aims to determine the optimal bandwidth by minimizing an estimate of the integrated squared error. The LSCV objective function can be expressed as


$$\begin{array}{c} LSCV(h)=\int \hat{f_{h}^{\;\text{2}}}(x)dx-\frac{\text{2}}{n}\sum_{i=\text{1}}^{n}{\hat{f}}_{h,-i}(x_{i}) \end{array}$$ (12)

where $\hat{f_{h}}$ denotes the KDE with bandwidth h, ${\hat{f}}_{h,-i}(x_{i})$ represents the leave-one-out density estimate at the i-th data point, and *n* is the sample size.

The first term $\int \hat{f_{h}^{\;\text{2}}}(x)dx$ measures the overall smoothness of the estimated density, while the second term evaluates the goodness-of-fit of the density estimate at the observed data points. In practical implementation, the LSCV criterion is evaluated numerically by approximating the integral term and the leave-one-out component over a range of candidate bandwidths. The optimal bandwidth is then selected by minimizing the LSCV objective function.

### 3.3. LSCV-B spline methods

To improve KDE accuracy, least-squares cross-validation (LSCV) is commonly adopted for bandwidth selection. In this study, the conventional Gaussian-kernel-based LSCV is extended by incorporating a B-spline kernel formulation, leading to an LSCV-B spline bandwidth selection strategy. By reconstructing the cross-validation criterion using compact-support kernel averaging and enforcing a minimum bandwidth constraint, the proposed approach provides a more stable and reliable bandwidth for residual density estimation, thereby improving the robustness of KDE-based interval prediction.

A first-order B-spline basis function with compact support is adopted and defined as


ϕ(x)=max(1−|x|,0) (13)

which provides favorable numerical stability and local adaptivity while avoiding oscillatory behavior associated with higher-order spline bases.

Based on the above basis function, a set of B-spline kernel averaging coefficients is constructed for a given candidate bandwidth *h* as


$$\begin{array}{c} \beta_{i}(h)=\frac{\text{1}}{n}\sum_{j=\text{1}}^{n}\phi (\frac{x_{j}-x_{i}}{h}) \end{array}$$ (14)

where $x_{i}$ denotes the i-th data point and *n* is the sample size.

Using these coefficients, the proposed LSCV-B objective function is defined as


$$\begin{array}{c} LSCV-B(h)=\frac{\text{1}}{\text{6}n}\sum_{i=\text{1}}^{n-\text{1}}(\beta_{i}^{\text{2}}+\text{4}\beta_{i}\beta_{i+\text{1}})-\frac{\text{2}}{nh}\sum_{i=\text{1}}^{n}\beta_{i} \end{array}$$ (15)

where the first term evaluates the smoothness of the estimated density, while the second term penalizes inadequate fit at the observed data points.

The optimal bandwidth is determined by minimizing this criterion over a predefined range of candidate bandwidths, with a minimum bandwidth constraint imposed to avoid under-smoothing and ensure numerical stability.

It should be noted that the B-spline basis is employed only in the construction of the bandwidth selection criterion. The final kernel density estimation is consistently performed using a Gaussian kernel with the selected bandwidth to ensure comparability with conventional ROT and LSCV based KDE methods.

### 3.4. Runoff interval prediction model framework

**Fig 2:**
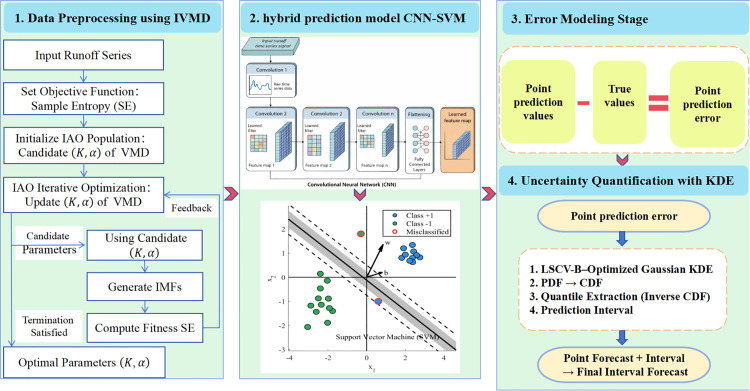
Overall framework of the proposed IVMD-CNN-SVM-LSCV-B runoff prediction model.

Due to the influence of various factors, runoff time series exhibit non-stationary and nonlinear characteristics. Therefore, this study proposes an improved point interval runoff prediction model IVMD-CNN-SVM-LSCV-B, which aims to enhance prediction accuracy and uncertainty quantification through the integration of multiple techniques. The overall structure is illustrated in Fig 2.

The modeling process is as follows:

**Step 1.** To effectively handle the nonlinear components in the original data, the algorithm-optimized IVMD is applied to preprocess the monthly runoff series. This step extracts more refined and representative IMFs, reduces noise interference, and enhances the intrinsic features of the data, providing a more accurate foundation for subsequent analysis.

**Step 2.** The decomposed IMFs are fed into the hybrid CNN-SVM model. In this model, CNN is responsible for feature extraction, automatically learning deep features of each IMF through its convolutional, pooling, and fully connected layers. This step enables efficient capture of both local and global patterns within the data, thus offering rich and informative feature representations for the SVM-based regression task.

**Step 3.** The point prediction results for runoff are obtained by summing the predicted values of all IMF components.

**Step 4.** The point prediction errors of the IVMD-CNN-SVM model are computed, and the LSCV-B method is used to determine the optimal bandwidth for Gaussian KDE to estimate the cumulative distribution function (CDF) of the errors. Based on the estimated CDF, the upper and lower bounds of the prediction interval are determined at a specified confidence level, forming the final comprehensive prediction interval. This step fully leverages the advantage of B-splines in capturing local features of the error distribution.

### 3.5. Prediction error evaluation metrics

#### 3.5.1. Deterministic point prediction evaluation metrics.

To quantitatively assess the deterministic point prediction performance of different models, four widely adopted evaluation metrics are employed, including Root Mean Squared Error (RMSE), Mean Absolute Percentage Error (MAPE), Coefficient of Determination (R^2^), and Kling Gupta Efficiency (KGE). RMSE and MAPE are used to characterize the overall magnitude and relative distribution of prediction errors, while R^2^ measures the goodness-of-fit between simulated and observed runoff. In addition, KGE is incorporated as a comprehensive hydrological performance index, as it simultaneously accounts for correlation, systematic bias, and variability discrepancies between predicted and observed runoff series, thereby providing a more holistic evaluation of model performance.

Lower RMSE and MAPE indicate higher point prediction accuracy, whereas higher R^2^ reflects stronger agreement between simulated and observed runoff. For KGE, values closer to 1 denote better overall hydrological consistency, while smaller values indicate increasing discrepancies in correlation, bias, or variability.


$$\left\{ \begin{array}{c} RMSE=\sqrt{\frac{\text{1}}{m}\sum_{t=\text{1}}^{m}{\left(y_{t}-{\hat{y}}_{t}\right)}^{\text{2}}}\\ \begin{array}{c} MAPE=\frac{\text{1}}{m}\sum_{t=\text{1}}^{m}\left|\frac{y_{t}-{\hat{y}}_{t}}{y_{t}}\right|\times \text{1}\text{0}\text{0}\%\\ R^{\text{2}}=\text{1}-\frac{\sum_{t=\text{1}}^{m}{\left(y_{t}-{\hat{y}}_{t}\right)}^{\text{2}}}{\sum_{t=\text{1}}^{m}{\left(y_{t}-{\overset{―}{y}}_{t}\right)}^{\text{2}}}\\ KGE=\text{1}-\sqrt{(r-\text{1}{)}^{\text{2}}+(\beta -\text{1}{)}^{\text{2}}+(\gamma -\text{1}{)}^{\text{2}}} \end{array} \end{array}\right.\;$$ (16)

where $y_{t}$ and ${\overset{―}{y}}_{t}$ represent the observed runoff and its mean value, respectively; ${\hat{y}}_{t}$ denotes the predicted runoff at time *t*; and *m* is the sample size. In the KGE formulation, *r* is the Pearson correlation coefficient between observed and predicted runoff series, β denotes the bias ratio, and γ represents the variability ratio.

#### 3.5.2. Uncertainty interval prediction evaluation metrics.

In addition to point prediction accuracy, a reliable runoff forecasting model should provide credible uncertainty quantification by accurately characterizing the distribution of prediction errors and the quality of prediction intervals. Therefore, both distribution fitting performance and interval reliability sharpness trade-offs need to be jointly evaluated. To assess the fitting accuracy of runoff prediction error distributions, three error-based metrics are adopted, including theMean Absolute Error (E_MAE_), Root Mean Squared Error (E_RMSE_), and Coefficient of Determination (E_R2_), as defined in Equation (17).


$$\left\{ \begin{array}{c} E_{MAE}=\frac{\text{1}}{n}\times \sum_{i=\text{1}}^{n}\left|c_{i}-{\hat{c}}_{i}\right|\\ E_{RMSE}=\sqrt{\frac{\text{1}}{n}\times \sum_{i=\text{1}}^{n}{\left(c_{i}-{\hat{c}}_{i}\right)}^{\text{2}}}\\ E_{R^{\text{2}}}=\text{1}-\frac{{SS}_{res}}{{SS}_{tot}} \end{array}\right.\;$$ (17)

where $c_{i}$ represents the empirical cumulative distribution function value, and ${\hat{c}}_{i}$ is the estimated CDF value. The E_R2_ is calculated using the sum of squared residuals (${SS}_{res}=\sum_{i=\text{1}}^{n}{\left(c_{i}-{\hat{c}}_{i}\right)}^{\text{2}}$) and the total sum of squares (${SS}_{tot}=\sum_{i=\text{1}}^{n}{\left(c_{i}-\overset{―}{c}\right)}^{\text{2}}$), where $\overset{―}{c}$ is the mean of $c_{i}$. The closer E_R2_ is to 1, E_MAE_ and E_RMSE_ are closer to 0, the better the fit, indicating higher distribution fitting performance.

For evaluating the performance of interval prediction methods, two key metrics are used: Prediction Interval Coverage Probability (PICP) and Prediction Interval Normalized Average Width (PINAW). PICP represents the proportion of actual observed values that fall within the prediction interval. For each time point, if the true value $y_{t}$ lies between the predicted lower bounds Lb(t) and upper bounds Ub(t), the count is incremented by 1.


$$\begin{array}{c} PICP=\frac{\text{1}}{m}\sum_{t=\text{1}}^{m}I\left\{Lb(t)\leq \;y_{t}\leq Ub(t)\right\} \end{array}$$ (18)

where *m* represents the sample size, and I(·) is an indicator function, which takes the value of 1 if the condition is true and 0 otherwise.

PINAW measures the average width of the prediction interval relative to the range of observed values:


$$\begin{array}{c} PINAW=\frac{\text{1}}{ms}\sum_{t=\text{1}}^{m}\left(Ub(t)-Lb(t)\right) \end{array}$$ (19)

where $s=y_{max}-y_{min}$ is the maximum and minimum values of the true observations, respectively.

A good interval prediction should have a high PICP and a small PINAW. However, in practical applications, reducing PINAW often comes at the cost of decreasing PICP. Therefore, this paper combines these two indicators using the harmonic mean to construct a comprehensive F-score:


$$F=\left\{ \begin{array}{c} \frac{\text{2}\times PICP\times \left(\frac{\text{1}}{PINAW}\right)}{PICP+\left(\frac{\text{1}}{PINAW}\right)},\;if\;PINAW>\text{0}\\ \;\text{0},\;otherwise \end{array}\right.\;$$ (20)

## 4. Case study data set

**Fig 3:**
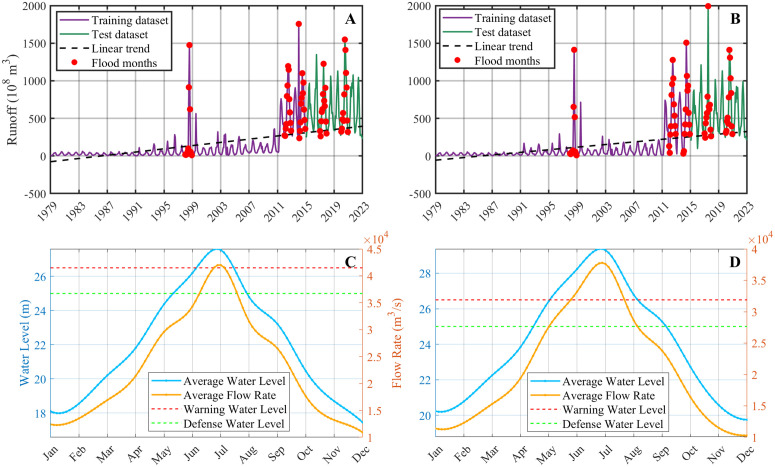
Monthly hydrological characteristics of the Yangtze River Basin at two stations. (A) Monthly runoff series at the Hankou station, including training and testing datasets, linear trend, and identified flood months. (B) Monthly runoff series at the Luoshan station, including training and testing datasets, linear trend, and identified flood months. (C) Monthly variations in multi-year mean water level and flow rate at the Hankou station, with warning and defense thresholds indicated. (D) Monthly variations in multi-year mean water level and flow rate at the Luoshan station, with warning and defense thresholds indicated.

This study focuses on the Yangtze River Basin in Hubei Province, China, and is based on 540 months of observed runoff data collected from January 1979 to December 2023 at the Hankou and Luoshan stations (Fig 3A and 3B). The monthly runoff series provides a long-term perspective on runoff variability under diverse hydrological conditions, covering both wet and dry seasons. Pronounced runoff peaks are evident in several years, notably 1998, 2016, and 2020, reflecting the occurrence of extreme flood events.

According to official flood control regulations of the Yangtze River Basin, the defense water level and warning water level are set at 25.0 m and 26.5 m, respectively. As illustrated in the multi-year monthly mean water level and flow rate variations (Fig 3C and 3D), the water level typically exceeds the warning level during July, corresponding to the main flood season. During this period, hydrological conditions become more complex and variable, which not only elevates flood risk but also poses substantial challenges for accurate runoff forecasting.

To facilitate model development and performance evaluation, the complete runoff dataset was divided into training and testing subsets using an 8:2 ratio, ensuring sufficient data for model learning while maintaining robust out-of-sample validation.

## 5. IVMD process for original runoff

### 5.1. Sensitivity and selection of VMD parameters

To assess the influence of VMD parameters on feature decomposition and predictive performance, a compact sensitivity analysis was conducted at the Luoshan station under a unified data preprocessing and evaluation protocol. To isolate the effect of VMD parameters, a standalone CNN model, rather than the CNN-SVM hybrid, was employed as the downstream predictor, with its architecture and training settings fixed and consistent with those described in Section 2.3.

A two-dimensional parameter grid was constructed by varying the number of modes *K* and the penalty factor α within predefined ranges, with K∈{5,6,7,8} and α∈{500,1000,1500, 2000}. For each (K,α) combination, VMD was first applied to decompose the runoff series, after which the resulting intrinsic mode functions were used to train the CNN model under identical architecture and training settings. The trained model was then evaluated on the test set, and the corresponding prediction performance was quantified using the test set RMSE.

**Fig 4:**
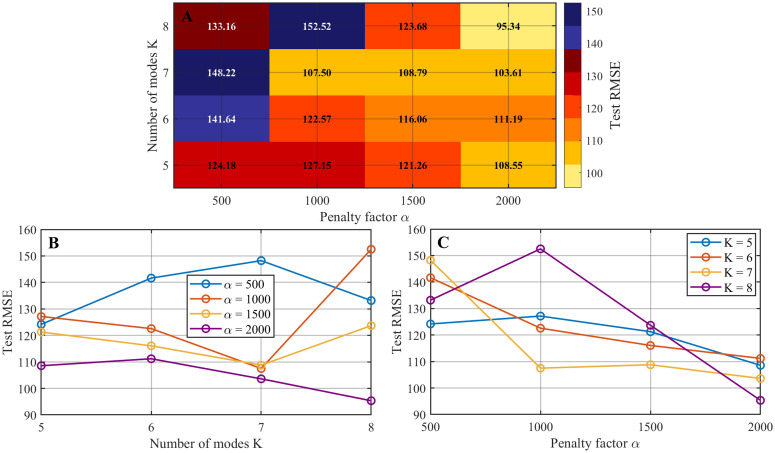
Sensitivity analysis of VMD parameters at the Luoshan station. (A) Heatmap of test RMSE values under different combinations of the number of modes *K* and the penalty factor α. (B) Test RMSE as a function of *K* with α fixed at different values. (C) Test RMSE as a function of α with *K* fixed at different values.

The sensitivity results are summarized in Fig 4, where Fig 4A presents a heatmap of test RMSE values across different combinations of the α and the number of modes *K*, offering an overview of the error distribution over the parameter space. Fig 4B and 4C show RMSE variations with respect to *K* at fixed α and with respect to α at fixed *K*, respectively.

These results indicate that both α and *K* have a substantial impact on decomposition effectiveness and downstream prediction accuracy, thereby motivating the need for adaptive parameter optimization.

### 5.2. IVMD optimization performance and decomposition results

To evaluate the effectiveness of entropy driven optimization in improving VMD decomposition quality, this subsection analyzes the optimization performance and decomposition results of the proposed IVMD framework. As introduced in Section 2.1.3, IVMD is employed to preprocess the original runoff series, where SE is adopted as the optimization objective to guide VMD parameter selection. By minimizing SE, the decomposition process is driven toward producing IMFs with enhanced regularity and reduced complexity.

To verify the optimization capability of the IAO, its performance is compared with several representative metaheuristic algorithms, including the Alpha (ALPHA) evolution algorithm, the Fata Morgana (FATA), the Escape (ESC), the weighted mean of vectors (INFO), and the Synergistic Swarm Optimization Algorithm (SSOA). All algorithms are implemented in MATLAB R2023b and executed under identical experimental settings with a population size of 15 and a maximum of 5 iterations.

To mitigate the influence of stochasticity, each algorithm is independently executed 20 times, and the stable results are reported. Runoff datasets from the Hankou and Luoshan stations are used to ensure a fair and robust comparison. The detailed optimization settings, including parameter search ranges and statistical results, are summarized in Table 2 **which** reports the comparative optimization performance of different algorithms in terms of SE minimization. It can be observed that the proposed IAO consistently achieves lower SE values at both stations, indicating superior optimization accuracy compared with the benchmark algorithms. In addition, the smaller dispersion of SE values obtained by IAO reflects its stronger stability under repeated runs.

**Table 2:** Optimized VMD parameters and objective function values at Hankou and Luoshan stations.

Station	Algorithm	Optimal *α*	Optimal *K*	Objective Value SE
**Hankou**	ALPHA	2320.82	10	0.007687
	SSOA	2496.18	10	0.007688
	FATA	2500.00	10	0.007689
	ESC	2118.79	10	0.007509
	INFO	2472.64	10	0.007670
	**IAO**	**2042.70**	**10**	**0.007419**
**Luoshan**	ALPHA	2161.61	10	0.007466
	SSOA	2055.09	10	0.007385
	FATA	2500.00	9	0.007655
	ESC	2082.66	10	0.007455
	INFO	2488.38	10	0.007636
	**IAO**	**2060.83**	**10**	**0.007316**

Note: VMD parameters were optimized within the ranges: K∈[3,10] and α∈[100,2500]. The number of modes *K* was rounded to the nearest integer for VMD decomposition. Bold values indicate the lowest objective function value at each site.

**Fig 5:**
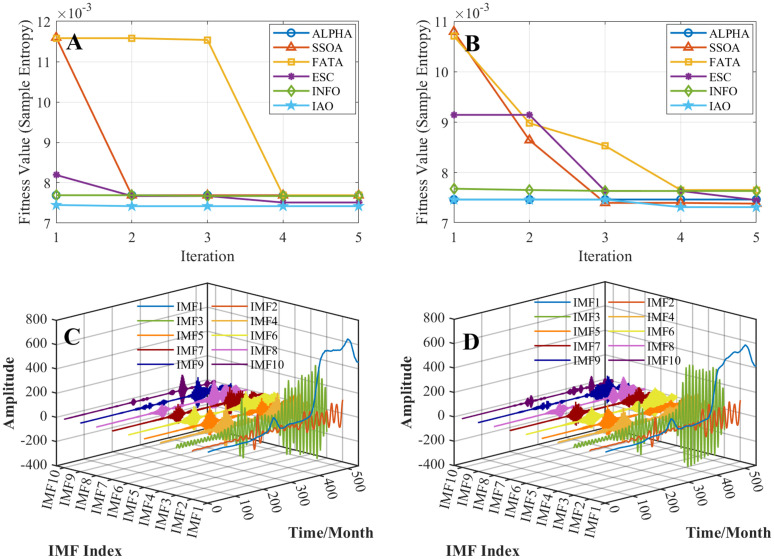
Optimization performance comparison of different algorithms for VMD and the corresponding IMF results. (A) Convergence curves of different optimization algorithms for VMD parameter optimization at the Hankou station. (B) Convergence curves of different optimization algorithms for VMD parameter optimization at the Luoshan station. (C) IMFs obtained by IVMD at the Hankou station. (D) IMFs obtained by IVMD at the Luoshan station.

Fig 5 compares the optimization performance of different algorithms for VMD parameter selection and their corresponding IVMD decomposition results. Fig 5A and 5B present the convergence curves of SE at the Hankou and Luoshan stations, respectively. All algorithms exhibit a consistent decline in fitness values with increasing iterations, confirming their ability to enhance VMD decomposition quality within the same optimization framework. Nevertheless, the proposed IAO converges faster and to significantly lower fitness levels than the benchmark algorithms at both stations. Using the optimal VMD parameters from IAO (Table 2), the IVMD framework decomposes the runoff series into multiple IMFs (Fig 5C and Fig 5D) with clearer scale separation, indicating improved decomposition quality.

**Fig 6:**
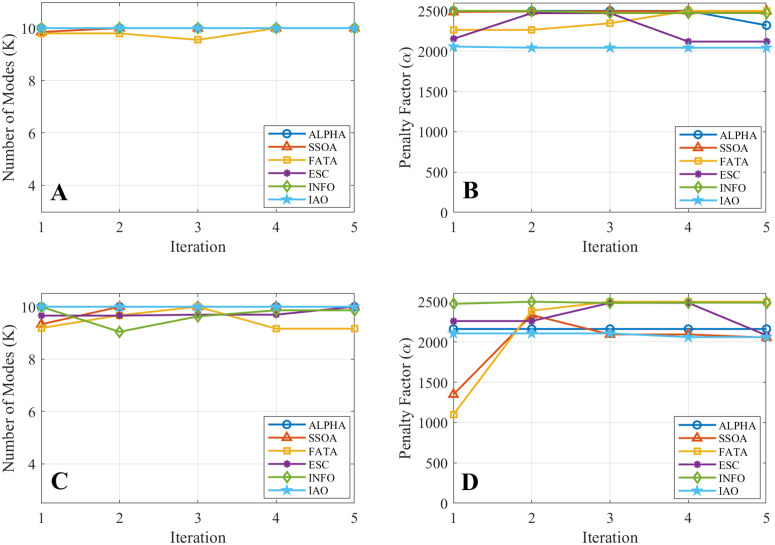
Iterative trajectories of optimized VMD parameters at two hydrological stations. (A) Iteration curve of the number of modes *K* at the Hankou station. (B) Iteration curve of the penalty factor *α* at the Hankou station. (C) Iteration curve of the number of modes *K* at the Luoshan station. (D) Iteration curve of the penalty factor *α* at the Luoshan station.

The iterative trajectories of the optimized VMD parameters are shown in Fig 6, where Fig 6A and 6B and Fig 6C and 6D correspond to the Hankou and Luoshan stations, respectively. Compared with the benchmark algorithms, IAO shows faster stabilization and smoother convergence of both the number of modes *K* and the penalty factor α, supporting its effectiveness in VMD parameter optimization.

### 5.3. Predicted results and analysis

#### 5.3.1. Deterministic point prediction of runoff.

To evaluate the effectiveness of the proposed IVMD framework for runoff point prediction, four single models (CNN, RVM, LSTM, and SVM) and three hybrid models (CNN-RVM, CNN-LSTM, and CNN-SVM) were examined at the Hankou and Luoshan stations. For comparison, all models were combined with conventional VMD and IVMD preprocessing, and their prediction performances were assessed on both the training and testing datasets using multiple deterministic evaluation metrics. All models were trained and evaluated using the same preprocessed datasets, where the original runoff series contained no missing values and were normalized to the range [0, 1] prior to model training.

The quantitative results for the training and testing datasets are summarized in Tables 3 and 4, respectively. Overall, hybrid models outperform single models under both preprocessing strategies, highlighting the advantage of model integration. Moreover, IVMD-based generally achieve lower RMSE and MAPE and higher R² and KGE than their VMD counterparts, confirming that automatic parameter optimization improves decomposition quality. Among all models, the proposed IVMD-CNN-SVM delivers the best overall performance at both stations. On the training set, it attains RMSE values of 7.0443 and 7.2899, with R^2^ exceeding 0.998 and KGE above 0.98. Compared with its VMD-based counterpart, the proposed IVMD-CNN-SVM model reduces RMSE by approximately 39.9% at Hankou and over 44.0% at Luoshan on the testing dataset, demonstrating enhanced statistical accuracy and hydrological reliability.

**Table 3:** Performance metrics of different models on the training dataset with VMD and IVMD preprocessing.

Model	Hankou				Luoshan			
	RMSE	MAPE	R^2^	KGE	RMSE	MAPE	R^2^	KGE
VMD-CNN	33.3696	0.5338	0.9684	0.9358	43.1038	0.7616	0.9402	0.8492
VMD-RVM	36.2323	0.6302	0.9627	0.9557	41.8453	0.6623	0.9613	0.8823
VMD-LSTM	33.1556	0.3423	0.9688	0.9316	29.8870	0.4365	0.9712	0.9209
VMD-SVM	34.8863	0.3529	0.9655	0.9226	30.6512	0.3803	0.9697	0.9148
VMD-CNN-RVM	35.7099	0.4049	0.9585	0.9181	42.2976	0.6871	0.9598	0.8895
VMD-CNN-LSTM	44.3168	0.4998	0.9443	0.8831	30.5667	0.3762	0.9699	0.9234
**VMD-CNN-SVM**	**19.7886**	**0.2707**	**0.9788**	**0.9588**	**18.1964**	**0.3049**	**0.9793**	**0.9682**
IVMD-CNN	31.2171	0.5939	0.9767	0.9426	35.2475	0.4908	0.9654	0.9010
IVMD-RVM	21.2734	0.5402	0.9892	0.9702	24.3598	0.4167	0.9835	0.9801
IVMD-LSTM	26.1084	0.8187	0.9837	0.9632	24.8519	0.4504	0.9828	0.9594
IVMD-SVM	33.0258	0.7369	0.9740	0.9259	27.4319	0.3705	0.9791	0.9299
IVMD-CNN-RVM	26.7892	0.7123	0.9751	0.9341	21.2469	0.4103	0.9858	0.9783
IVMD-CNN-LSTM	26.7575	0.8126	0.9829	0.9249	19.7454	0.6844	0.9892	0.9085
**IVMD-CNN-SVM**	**7.0443**	**0.3227**	**0.9988**	**0.9832**	**7.2899**	**0.2626**	**0.9985**	**0.9879**

Note: Bold values indicate the best performance among all compared models on the training dataset.

**Table 4:** Performance metrics of different models on the testing dataset with VMD and IVMD preprocessing.

Model	Hankou				Luoshan			
	RMSE	MAPE	R^2^	KGE	RMSE	MAPE	R^2^	KGE
VMD-CNN	75.7379	0.1458	0.9025	0.7730	74.2144	0.1479	0.9113	0.7779
VMD-RVM	65.2698	0.0919	0.9276	0.9395	65.9988	0.1084	0.9298	0.9438
VMD-LSTM	65.9851	0.1046	0.9260	0.8199	55.5502	0.0818	0.9503	0.9588
VMD-SVM	51.2368	0.0785	0.9554	0.8951	50.9342	0.0748	0.9582	0.9557
VMD-CNN-RVM	56.8905	0.0841	0.9449	0.8635	67.2270	0.1227	0.9273	0.8880
VMD-CNN-LSTM	64.5629	0.0851	0.9291	0.8494	53.1995	0.0721	0.9544	0.8839
**VMD-CNN-SVM**	**29.9064**	**0.0546**	**0.9783**	**0.9505**	**35.6383**	**0.0641**	**0.9795**	**0.9682**
IVMD-CNN	63.9962	0.1045	0.9421	0.8879	62.8769	0.1010	0.9497	0.8590
IVMD-RVM	42.8570	0.0728	0.9740	0.9594	63.4519	0.1155	0.9488	0.9629
IVMD-LSTM	47.5513	0.0893	0.9681	0.9448	56.3053	0.1035	0.9597	0.9752
IVMD-SVM	49.1515	0.0711	0.9658	0.9455	50.9769	0.0835	0.9670	0.9696
IVMD-CNN-RVM	50.2599	0.0860	0.9643	0.9501	46.5556	0.0773	0.9724	0.9653
IVMD-CNN-LSTM	51.249	0.0766	0.9628	0.8718	56.8443	0.0980	0.9589	0.9307
**IVMD-CNN-SVM**	**17.9749**	**0.0307**	**0.9954**	**0.9713**	**19.9491**	**0.0318**	**0.9949**	**0.9845**

Note: Bold values indicate the best performance among all compared models on the testing dataset.

**Fig 7:**
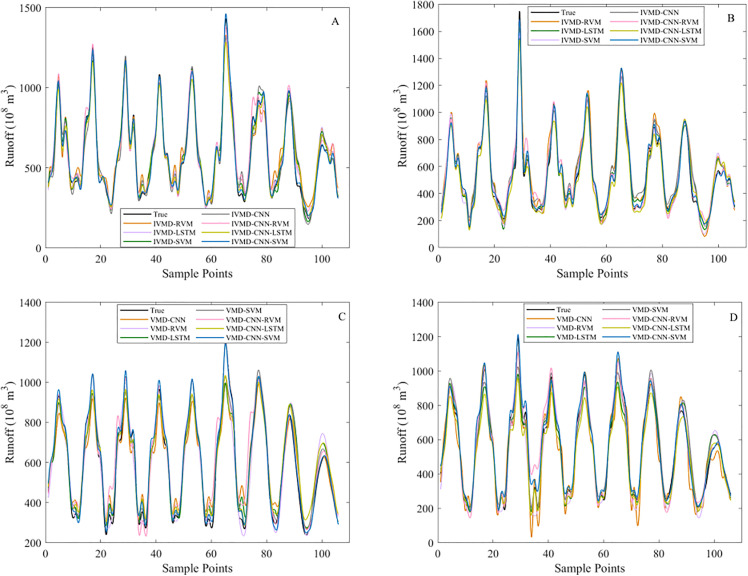
Comparison of runoff point prediction results under IVMD and VMD preprocessing. (A) Runoff point prediction curve at the Hankou station using an IVMD-based model. (B) Runoff point prediction curve at the Luoshan station using an IVMD-based model. (C) Runoff point prediction curve at the Hankou station using a VMD-based model. (D) Runoff point prediction curve at the Luoshan station using a VMD-based model.

Fig 7 compares the runoff point prediction results on the testing dataset obtained using different preprocessing strategies. Specifically, Fig 7A and 7B show the predicted and observed runoff series at the Hankou and Luoshan stations based on IVMD preprocessing, while Fig 7C and 7D present the corresponding results using VMD preprocessing.

In general, the IVMD-based results show better agreement with the observed runoff series at both stations. Compared with VMD preprocessing, IVMD preprocessing produces predictions that more closely follow the temporal evolution of runoff, with smaller deviations in peak values and smoother transitions between adjacent periods.

**Fig 8:**
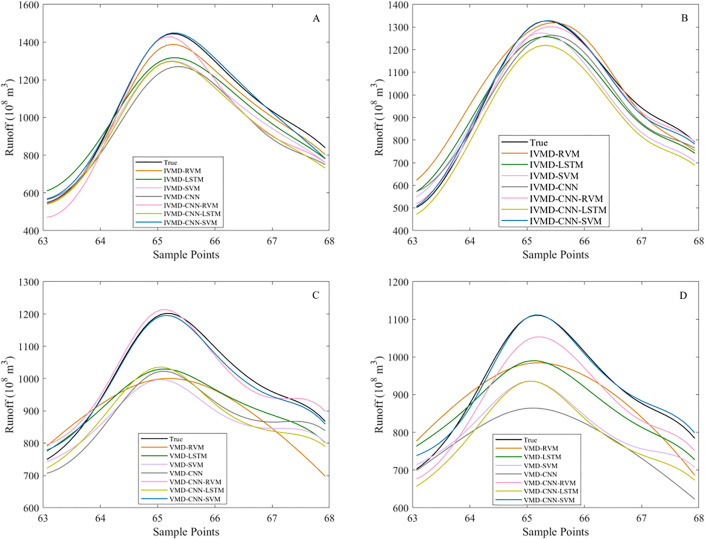
Local enlarged comparison of runoff point predictions under IVMD and VMD preprocessing. (A) Local enlarged runoff point prediction curve at the Hankou station using an IVMD-based model. (B) Local enlarged runoff point prediction curve at the Luoshan station using an IVMD-based model. (C) Local enlarged runoff point prediction curve at the Hankou station using a VMD-based model. (D) Local enlarged runoff point prediction curve at the Luoshan station using a VMD-based model.

To further examine model performance during critical periods, Fig 8 presents local enlarged views of the runoff point prediction results. Fig 8A and 8B show the local comparisons at the Hankou and Luoshan stations using IVMD-based models, while Fig 8C and 8D display the corresponding results obtained with VMD preprocessing.

At both stations, the IVMD-CNN-SVM model shows a closer match to the observed runoff in peak regions and during rapid rising and falling stages. Compared with VMD-based results, IVMD-based predictions show smaller deviations near turning points and smoother transitions around peak values, indicating better local fitting performance. Overall, the local enlarged views further illustrate the advantage of IVMD in capturing short-term variations and peak dynamics in runoff point prediction.

#### 5.3.2. Runoff error distribution and KDE based performance evaluation.

Accurate characterization of prediction error distributions is essential for enhancing runoff interval forecasting. To examine the effect of bandwidth selection in KDE-based interval prediction, three representative methods ROT, LSCV, and LSCV-B are evaluated based on the IVMD-CNN-SVM model at the Hankou and Luoshan stations, with performance assessed through (i) error distribution fit (E_MAE_, E_RMSE_, E_R2_) and (ii) 90% interval prediction quality (PICP, PINAW, F). The results are summarized in Table 5.

**Table 5:** KDE bandwidth comparison for IVMD-CNN-SVM: error fit (E_MAE_, E_RMSE_, E_R2_) and 90% interval metrics (PICP, PINAW, F) at two stations.

Station	Model	E_MAE_	E_RMSE_	E_R2_	PICP	PINAW	F
**Hankou**	IVMD-CNN-SVM-ROT	0.1106	0.1350	0.9789	0.9340	**0.0570**	1.7734
	IVMD-CNN-SVM-LSCV	0.1283	0.1599	0.9704	0.9434	0.0586	1.7879
	**IVMD-CNN-SVM-LSCV-B**	**0.0709**	**0.0935**	**0.9899**	**0.9528**	0.0632	**1.7975**
**Luoshan**	IVMD-CNN-SVM-ROT	0.1279	0.1793	0.9598	0.9151	0.0410	1.7640
	IVMD-CNN-SVM-LSCV	0.1123	0.1524	0.9710	0.9151	**0.0403**	1.7651
	**IVMD-CNN-SVM-LSCV-B**	**0.0858**	**0.1059**	**0.9860**	**0.9529**	**0.0437**	**1.8295**

Note: Bold values indicate the best performance among different KDE bandwidth selection methods for each station. E_MAE_, E_RMSE_, and E_R2_ measure the goodness-of-fit of the KDE to the empirical prediction error distribution; PICP, PINAW, and F evaluate the 90% prediction intervals.

As summarized in Table 5, the LSCV-B method consistently provides the most accurate error distribution fitting for the IVMD-CNN-SVM model at both stations. At the Hankou station, LSCV-B reduces E_MAE_ and E_RMSE_ by approximately 35.9% and 30.7% relative to the ROT method, and by 44.7% and 41.5% compared with the conventional LSCV approach, respectively. At the Luoshan station, the corresponding reductions achieved by LSCV-B reach 32.9% and 40.9% relative to ROT, and 23.6% and 30.5% compared with LSCV. Moreover, the E_R2_ values obtained using LSCV-B are consistently closest to unity at both stations, indicating a superior goodness-of-fit of the estimated error distributions.

**Fig 9:**
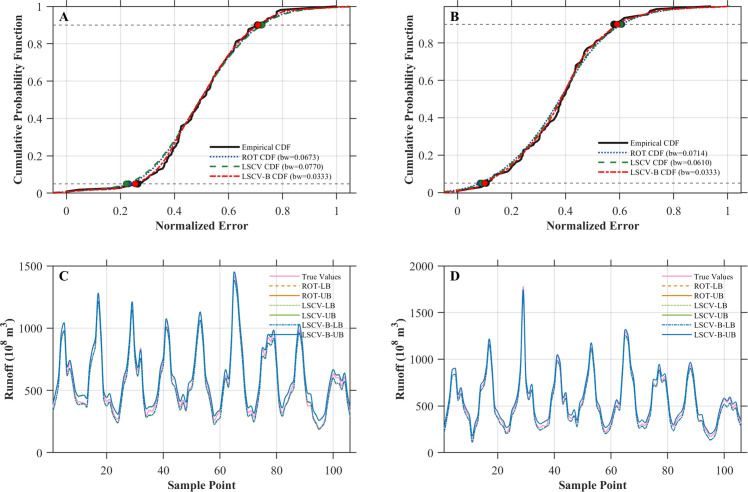
Comparison of interval prediction performance using different KDE bandwidth selection methods. (A) Empirical and fitted CDFs of normalized prediction errors at the Hankou station. (B) Empirical and fitted CDFs of normalized prediction errors at the Luoshan station. (C) Prediction intervals obtained using different KDE bandwidth selection methods at the Hankou station. (D) Prediction intervals obtained using different KDE bandwidth selection methods at the Luoshan station.

The CDF comparisons in Fig 9A and 9B indicate that the LSCV-B method provides a closer fit to the empirical error distribution than ROT and LSCV, particularly around the 5% and 90% quantiles. Correspondingly, Fig 9C and 9D show that LSCV-B yields relatively tighter prediction intervals that more accurately follow observed runoff fluctuations, while ROT and LSCV tend to produce wider intervals despite achieving comparable coverage.

## 6. Discussion and conclusion

### 6.1. Discussion

To further assess the robustness of the proposed IVMD-CNN-SVM-LSCV-B framework, its interval prediction performance is systematically compared with that of the Bootstrap-based approach under multiple confidence levels. The Bootstrap method, which constructs prediction intervals by repeatedly resampling the empirical prediction errors, is employed as a representative data-driven benchmark for uncertainty quantification.

The quantitative comparison results at the 95%, 90%, and 85% confidence levels for both Hankou and Luoshan stations are summarized in Table 6. Overall, the IVMD-CNN-SVM-LSCV-B model exhibits a more favorable balance between coverage reliability and interval compactness across all confidence levels and stations. Specifically, at both stations, the LSCV-B based intervals consistently achieve coverage probabilities close to or exceeding the nominal confidence levels, while maintaining relatively narrow interval widths, as reflected by lower PINAW values and higher composite F scores.

**Table 6:** Comparison of interval prediction performance between IVMD-CNN-SVM-LSCV-B and Bootstrap methods under different confidence levels.

Station	Model	Confidence Level	PICP	PINAW	F
**Hankou**	IVMD-CNN-SVM-Bootstrap	95%	0.9528	0.0613	1.8006
		90%	0.8868	0.0513	1.6963
		85%	0.8396	0.0452	1.6179
	IVMD-CNN-SVM-LSCV-B	95%	0.9623	0.0779	1.7904
		90%	0.9528	0.0632	1.7975
		85%	0.9057	0.0547	1.7259
**Luoshan**	IVMD-CNN-SVM-Bootstrap	95%	0.9623	0.0439	1.8466
		90%	0.8962	0.0399	1.7306
		85%	0.8396	0.0325	1.6346
	IVMD-CNN-SVM-LSCV-B	95%	0.9717	0.0532	1.8478
		90%	0.9528	0.0437	1.8295
		85%	0.8962	0.0378	1.7337

In contrast, the Bootstrap method shows noticeable performance degradation as the confidence level increases. At the 95% confidence level, Bootstrap-based intervals at both stations tend to under-cover the observations, indicating difficulty in capturing the tail behavior of prediction errors through resampling alone. This limitation is further manifested by comparatively wider or less stable intervals at lower confidence levels, leading to reduced overall reliability in interval estimation.

**Fig 10:**
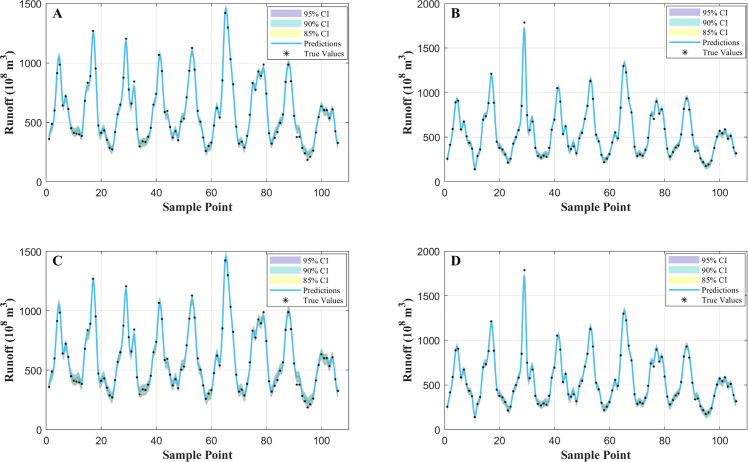
Comparison of interval prediction performance between Bootstrap and LSCV-B methods at two stations. (A) Bootstrap-based prediction intervals at the Hankou station. (B) Bootstrap-based prediction intervals at the Luoshan station. (C) LSCV-B-based prediction intervals at the Hankou station. (D) LSCV-B-based prediction intervals at the Luoshan station.

The corresponding interval prediction curves are shown in Fig 10. The Bootstrap-based prediction intervals for the Hankou and Luoshan stations are presented in Fig 10A and Fig 10B, respectively, whereas the intervals obtained using the LSCV-B method under the same confidence levels are shown in Fig 10C and Fig 10D. Overall, the LSCV-B intervals appear smoother and track the observed runoff dynamics more consistently. During rapid fluctuations and peak runoff periods, the LSCV-B intervals adjust more steadily to changes in uncertainty, while the Bootstrap intervals occasionally become overly wide or locally irregular.

These observations suggest that KDE-based error distribution modeling with LSCV-B bandwidth selection can provide a more stable characterization of predictive uncertainty than conventional Bootstrap resampling in this setting, leading to more practically interpretable interval forecasts across multiple confidence levels.

### 6.2. Conclusion

This paper presents a hybrid runoff interval prediction model, IVMD-CNN-SVM-LSCV-B, which integrates IVMD, CNN, SVM, and the LSCV-B spline-based kernel density estimation method, with parameter optimization guided by the IAO. The proposed framework demonstrates strong performance in both point and interval prediction, particularly in modeling complex and nonlinear runoff processes.

Experimental results demonstrate the following:

i Compared with the VMD-CNN-SVM benchmark on the testing dataset, the proposed IVMD-CNN-SVM model reduces RMSE by 39.9% at Hankou and 44.0% at Luoshan, while MAPE decreases by 43.8% and 50.4%, respectively. These improvements highlight the effectiveness of entropy-guided decomposition combined with hybrid deep learning.
ii The IVMD-CNN-SVM model consistently outperforms all baseline models at both stations, confirming its capability to extract informative features through coordinated decomposition and learning.
iii The LSCV-B method further enhances interval prediction by enabling accurate error density estimation, producing narrower confidence intervals while maintaining high coverage probability, and showing good adaptability to skewed and multimodal error distributions.

Overall, the proposed framework provides a reliable solution for runoff point-interval prediction within the studied hydrological context. By adopting a multi-stage modeling strategy, the framework achieves substantial improvements in predictive accuracy and uncertainty quantification, making it particularly suitable for offline analysis and decision-support applications. While the present evaluation focuses on stations within a single river basin, extending the framework to broader hydrological settings, incorporating additional process-related information, and further improving computational efficiency may enhance its robustness, applicability, and potential for wider operational use. These aspects will be explored in future research.
